# Using Citizen Science Data to Model the Distributions of Common Songbirds of Turkey Under Different Global Climatic Change Scenarios

**DOI:** 10.1371/journal.pone.0068037

**Published:** 2013-07-03

**Authors:** Moris Abolafya, Ortaç Onmuş, Çağan H. Şekercioğlu, Raşit Bilgin

**Affiliations:** 1 Institute of Environmental Sciences, Boğaziçi University, Istanbul, Turkey; 2 Department of Biology, Natural History Museum Research and Application Center, Ege University, Izmir, Turkey; 3 Department of Biology, University of Utah, Salt Lake City, Utah, United States of America; 4 KuzeyDoğa Derneği, Kars, Turkey; Plymouth University, United Kingdom

## Abstract

In this study, we evaluated the potential impact of climate change on the distributions of Turkey’s songbirds in the 21st century by modelling future distributions of 20 resident and nine migratory species under two global climate change scenarios. We combined verified data from an ornithological citizen science initiative (www.kusbank.org) with maximum entropy modeling and eight bioclimatic variables to estimate species distributions and projections for future time periods. Model predictions for resident and migratory species showed high variability, with some species projected to lose and others projected to gain suitable habitat. Our study helps improve the understanding of the current and potential future distributions of Turkey’s songbirds and their responses to climate change, highlights effective strategies to maximize avian conservation efforts in the study region, and provides a model for using citizen science data for biodiversity research in a large developing country with few professional field biologists. Our results demonstrate that climate change will not affect every species equally in Turkey. Expected range reductions in some breeding species will increase the risk of local extinction, whereas others are likely to expand their ranges.

## Introduction

Large-scale anthropogenic climatic change has been documented since the mid-20th century and average global temperature has increased by 0.7°C over the past 100 years [1]. Although climate has been continuously changing throughout the planet’s history, forcing species to adapt or to go extinct, the current pace of climate change is particularly threatening to biodiversity because climate is changing at a rate much faster than most species can adapt [2], [3]. Recent, rapid climate change and future projections of human-induced climate change indicate that biological diversity and habitats worldwide are likely to be drastically affected in the near future [4]–[7].

Climate change has vital implications for biodiversity. It has an effect on a wide variety of organisms with diverse geographical distributions, affecting their physiology, distributions, phenology, and behavior. Furthermore, climate change can render species’ ranges unsuitable and force populations to move from their current locations to new and unoccupied areas. During this transition, populations may become highly fragmented and local extinctions may occur [4], [5], [8]–[10]. However, species do not necessarily ‘go without a fight’; they may adapt themselves to these changes either ecologically [3] or evolutionarily [7], [11], [12]. Nevertheless, species and/or populations that cannot exhibit adequate levels of adaptation will probably go extinct, either locally or globally. Species with limited climatic tolerance and specialized habitat requirements, limited dispersal abilities and thermal physiology are more likely to be affected this way [2], [7], [9].

Birds have been increasingly documented to be shifting their elevational ranges and colonizing new localities due to climate change [13]–[15]. For instance, Tingley *et al*. [16] found that 48 out of the 53 species in the Sierra Nevada mountains of California shifted their average bioclimatic range over 100 years. Phenological shifts in relation to climate change have also been documented. For instance, some populations of songbirds (passerines, order Passeriformes) have decreased as a consequence of climate change, since the phenology of their main food supplies such as flowers, seeds and insects during breeding has advanced faster than birds’ breeding periods, resulting in phenological mismatch [3], [7], [17]–[20]. Other changes associated with climate change include changes in abundance, interactions with habitat fragmentation, and changes in the timing of lifecycle events, such as hibernation and estivation [21], [22]. The interactions of these effects further contribute to disruptions in species’ population dynamics, potentially leading to additional changes in distribution [22], [23].

As a consequence of its geographic, topographic, and climatic diversity, Turkey hosts exceptionally rich biodiversity that is increasingly threatened [24], [25]. Three out of 34 “biodiversity hotspots” in the world meet in Turkey: Mediterranean, Caucasus, and Irano-Anatolian [25]. Turkey is also home to a range of terrestrial biomes, including Mediterranean forests, woodlands, and shrublands, temperate broadleaf and mixed forests, temperate coniferous forests, montane grasslands and shrublands, temperate grasslands, semi-deserts, and xeric shrublands [24], [26].

Such biome diversity contributes to high plant and animal diversity in Turkey, and birds provide a good example. Anatolia hosts a diverse bird fauna of 470 species that is steadily increasing with more research [25]. Turkey is also an important region for the study of geographical variation in the bird populations of the Western Palearctic region [27], [28]. Even though the distributions of most bird species in Turkey are relatively well known, to our knowledge there has not been a study on how climatic change will shape the future distributions of Turkey’s birds. The effects of climate change on the distributions of lesser-known bats have been modelled [29], and we have substantially more data for birds. In this study, we evaluate the potential impact of climate change on the distributions of Turkey’s songbirds in the 21st century, using data from Turkey’s most extensive citizen science project [30]. We have created species richness maps based on songbirds’ current distributions and projected their future distributions to understand the trends in resident and migratory species’ ranges over time. In addition to improving our understanding of the current and future distributions of Turkey’s songbirds and their responses to climate change, our modeling approach also helps highlight effective strategies to maximize avian conservation efforts in the study region.

Our study is also an excellent model for using high-quality citizen science biodiversity data for understanding how species’ distributions will respond to global change [31]. As funding for biodiversity research becomes increasingly scarce and amateur naturalists’ numbers grow rapidly, data collected by citizen scientists are becoming increasingly important for global change research [31]–[33]. This is particularly the case for ornithology, where millions of birdwatchers have made significant contributions to avian research for centuries [3], [34]. The role of citizen scientists is especially important in developing countries like Turkey where there are few professional ornithologists, no funding for long-term bird monitoring programs, and a rapidly growing number of experienced and dedicated amateur ornithologists. Our study is based on a citizen ornithology program in Turkey (www.kusbank.org) [30] that is similar to e-bird (www.ebird.org) [32]. Our findings show the value of citizen science data for global change research and provide a good model for similar studies, particularly in biodiverse developing countries with few professional field biologists and inadequate biodiversity research [7].

## Methods

### Study Species

We modelled the distributions of resident (non-migratory) and migratory songbirds, using presence-only data from the Kuşbank database (“Birdbank”, [30]), which holds over 430,000 records of the birds of Turkey. These publicly-available data consist of observations collected by ornithologists and amateur birdwatchers. Bird records are publicly seen and validated by the administrators and the rest of Turkey’s birdwatching community. Providers of unusual records are asked to provide detailed supporting information to be reviewed by experts, in a manner similar to Cornell University’s e-bird [32].

When we undertook our analyses, the number of songbird species submitted to Kuşbank was 151 and the corresponding number of records was over 152,000. Among these, a total of 57,149 records corresponding to 29 (20 resident/non-migratory and nine summer migratory) songbird bird species were selected for modelling. We used the following criteria for selecting species for analysis.

We limited our analysis to species with sufficient occurrence data. Only species with more than 25 data points were chosen for modeling. 15 species were excluded due to this criterion. Another requirement was for the existing records of a species to be relatively evenly distributed throughout the study area. Almost half of the songbird species and their records are artificially concentrated in some popular and frequently visited sites in or around the cities. These species had to be excluded from the analyses, as the use of these records would create a strong bias. After this initial round of species selection, we validated the data for possible misidentification and tried to reduce possible biases. An assumption of our analyses is that because songbirds are mobile species, each species has the ability to move independently between different areas. We first homogenized the sampling effort by removing the records from existing local Breeding Bird Surveys (BBS) and ringing (banding) stations. Therefore, we reduced a possible source of bias due to excessive number of records at some sites. For migratory species, we excluded the records outside the breeding season (April 15–July 30), as these migration records likely represent non-breeding ranges. To cope with excessively harsh climatic conditions during winter, some resident bird species can exhibit medium- to large-scale changes in their ranges, exhibit nomadism, and roost in other locations than those they prefer during breeding. Therefore, all winter observations, between December 1st and January 31th, were excluded from our analyses. Finally, the records of three cryptic species (Olive-tree Warbler *Hippolais olivetorum*, Greenish Warbler *Phylloscopus trochiloides*, and Mountain Chiffchaff *Phylloscopus sindianus*) were validated based on the observations of experienced birdwatchers only. These cryptic species are difficult to differentiate and require extensive experience to identify. After selecting the data to minimize bias, models were run for the remaining species, and the results were compared with already known species distribution maps [27], [28]. Following visual inspection, the species whose models did not match their known distributions were excluded from the subsequent analyses.

Based on the elimination procedure outlined above, 40,782-point records of 29 species (38,447 for resident species and 2335 for migratory species) were used for the subsequent analyses (Table 1, Table 2).

**Table 1:** Resident species names and number of presence records.

Scientific name	Common name	Presence Records
*Melanocorypha calandra*	Calandra Lark	794
*Calandrella brachydactyla*	Greater Short-toed Lark	262
*Galerida cristata*	Crested Lark	2922
*Pycnonotus xanthopygos*	White-spectacled Bulbul	415
*Turdus merula*	Eurasian Blackbird	3685
*Parus ater*	Coal Tit	890
*Parus caeruleus*	Blue Tit	1479
*Parus major*	Great Tit	3590
*Sitta europaea*	Wood Nuthatch	388
*Sitta tephronota*	Eastern Rock Nuthatch	198
*Sitta neumayer*	Western Rock Nuthatch	1024
*Garrulus glandarius*	Eurasian Jay	2407
*Pica pica*	Black-billed Magpie	3904
*Corvus monedula*	Eurasian Jackdaw	2237
*Corvus corone palescens*	Hooded Crow	4234
*Corvus corax*	Common Raven	936
*Passer domesticus*	House Sparrow	5097
*Passer montanus*	Eurasian Tree Sparrow	177
*Petronia petronia*	Rock Sparrow	533
*Carduelis carduelis*	European Goldfinch	3275
**Total**		**38447**

**Table 2:** Migratory species names and number of presence records.

Scientific name	Common name	Presence Records
*Hirundo daurica*	Red-rumped Swallow	700
*Erythropygia galactotes*	Rufous-tailed Scrub-robin	669
*Hippolais olivetorum*	Olive-tree Warbler	65
*Sylvia cantillans*	Subalpine Warbler	35
*Phylloscopus trochiloides*	Greenish Warbler	25
*Phylloscopus sindianus*	Mountain Chiffchaff	29
*Lanius nubicus*	Masked Shrike	411
*Carpodacus erythrinus*	Common Rosefinch	260
*Emberiza caesia*	Cretzschmar's Bunting	141
**Total**		**2335**

### Climatic Data (Current and Future)

Average monthly climate data (precipitation, minimum, and maximum temperature) were retrieved from www.worldclim.org with a 2.5 arc-minutes grid resolution (∼5 km×5 km resolution) for the current and future time periods (2020, 2050, and 2080). Nineteen bioclimatic variables were generated for the current and future conditions by using average monthly climate data, and the software ArcGIS v. 10.0 and DivaGIS [34], [35]. Hadley Centre Coupled Model version 3 (HADCM3) was used as our global climate model to simulate the impact of the IPPC third assessment storylines A2a and B2a [36] on future climate conditions. All these layers were masked on an area between 26°E and 45°E and 36°N and 42°N, which includes the entire territory of Turkey.

A series of a correlation tests were conducted and correlation matrices were generated for all 19 bioclimatic variables using the software ENMTools [37] in order to eliminate bioclimatic variables that include redundant information for the models. This software helps assess the identity and similarity of ecological niches or variables. Pearson correlation coefficient of 0.75 was used as a threshold to pair highly correlated bioclimatic variables [38]. Eight biologically meaningful variables were chosen as a result of this treatment, and were used in all subsequent analyses (Table 3). For migratory species, instead of the two variables BIO1 (Annual Mean Temperature) and BIO14 (Precipitation of Driest Month), their correlated variables BIO10 (Mean Temperature of Warmest Quarter) and BIO18 (Precipitation of Warmest Quarter), were used respectively as they are more directly associated with the warmer summer season when these migratory species are actually present. Using these eight bioclimatic variables, projections for 2020, 2050, and 2080 time periods were produced.

**Table 3:** The order of AUC values, variable contributions, and indication times for each variable that show the most relevancy for a resident species.

Bioclimatic variables	Average AUC without	Sum of Contribution	Number of species
Temperature Seasonality (BIO4)	0.88	491.25	7
Annual mean temperature (BIO1)	0.88	460.52	8
Precipitation of seasonality (BIO15)	0.88	260.72	1
Annual Precipitation (BIO12)	0.88	228.61	3
Precipitation of Driest Month (BIO14)	0.88	203.26	1
Mean temperature of wettest quarter (BIO8)	0.88	167.64	0
Mean diurnal range in temperature (BIO2)	0.89	108.02	0
Isothermality (monthly/annual) (BIO3)	0.89	79.98	0

### Modeling

The climate envelope was built with the data on species and climate. The locations of particular species were used as dependent variables, and various climatic data were used as independent variables. Subsequently, the relationship between a species’ known distribution and various climatic variables were quantified.

MAXENT version 3.2.19 (www.cs.princeton.edu/~schapire/maxent), a maximum entropy modelling method, was used to estimate species actual geographic distributions based on presence data. Maxent is a general-purpose algorithm that can generate predictions or inferences from an incomplete set of information. Predictive maps generated by Maxent express suitability of each grid cell as a function of the environmental variables in that grid cell [39]. The models were run with the default settings of Maxent: auto features, 10,000 background points, regularization multiplier = 1.0, maximum iterations = 1000 and convergence threshold = 0.00005. To assess the importance of each predictor variable, the jackknifing procedures were implemented in Maxent. The area under the receiver operator curve (AUC) was used to assess overall model performance, where an AUC score of 0.5 indicates a random prediction, and a score of 1 a perfect prediction. In order to assess the robustness of the model to sampling variation, 25% of the presence data from the original dataset were chosen randomly to test the model while the remaining 75% were used in training it.

### Integrating the Models

The binary presence/absence maps of each species were produced for the time periods current, 2020, 2050 and 2080, using the Maxent probabilities and 10 percentile training presence logistic thresholds as a cut-off parameter [40]. In order to produce species richness (biodiversity) maps for each modelled scenario and time period, all binary maps from each species’ biogeographic group were combined. ArcGIS v. 10.0 software and scripts prepared in Python programming language [41] were used while combining the maps. This made it possible to construct maps depicting the possible loss or gain of species in a particular area (pixel) over time.

### Changes in Occupied Area Over Time

Presence or absence of a species in a cell is indicated by 1 or 0, respectively. In order to create presence overlap maps for different time periods, current data were multiplied by predicted future data for each cell. In these maps, the locations where a species is present both in current and predicted time periods is indicated by 1. The percentage of presence overlap was calculated by dividing the count of locations where species were found to be present in predicted future presence maps by the presence counts in the current maps. Python scripts in ArcGIS were also used for making these calculations [41]. In addition, zonal centroids of the presence models were calculated using ArcGIS 10.0 both in current and predicted time periods. The analyses approximate the geometry of each zone by creating an ellipse fixed at the centroid of each zonal spatial shape and include the calculation of the eigenvalues and eigenvectors of each zone.

## Results

All AUC values, which determine model performance, were 0.88 or higher (Table 3) and averaged 0.91 in all 20 models, indicating that the model had good predictive power. ROC plots predicted very similar AUC values between training and test data, even though the values for resident species were slightly lower for the test data (0.89). In addition to the average training, AUC values of the model including all eight variables were higher than those excluding one of the eight variables. This comparison also shows that the predictive ability of the model decreases if any of the eight variables are excluded. While evaluating the results for the summer migrants, ROC plots predicted higher AUC values between training (0.95) and test data (0.93) when compared to the resident species (Table 4), with an average AUC value of 0.95 in all nine models.

**Table 4:** The order of AUC values, variable contributions, and indication times for each variable that show the most relevancy for a migratory species.

Bioclimatic variables	AverageAUC without	Sum ofContribution	Number of species
Mean Temperature of Warmest Quarter(BIO10)	0.93	231.67	3
Precipitation of seasonality (BIO15)	0.93	221.53	3
Precipitation of Warmest Quarter (BIO18)	0.93	144.46	3
Temperature Seasonality (BIO4)	0.93	106.77	0
Mean temperature of wettest quarter (BIO8)	0.92	33.15	0
Annual Precipitation (BIO12)	0.93	23.33	0
Mean diurnal range in temperature (BIO2)	0.93	22.48	0
Isothermality (monthly/annual) (BIO3)	0.93	16.59	0

**Figure 1:**
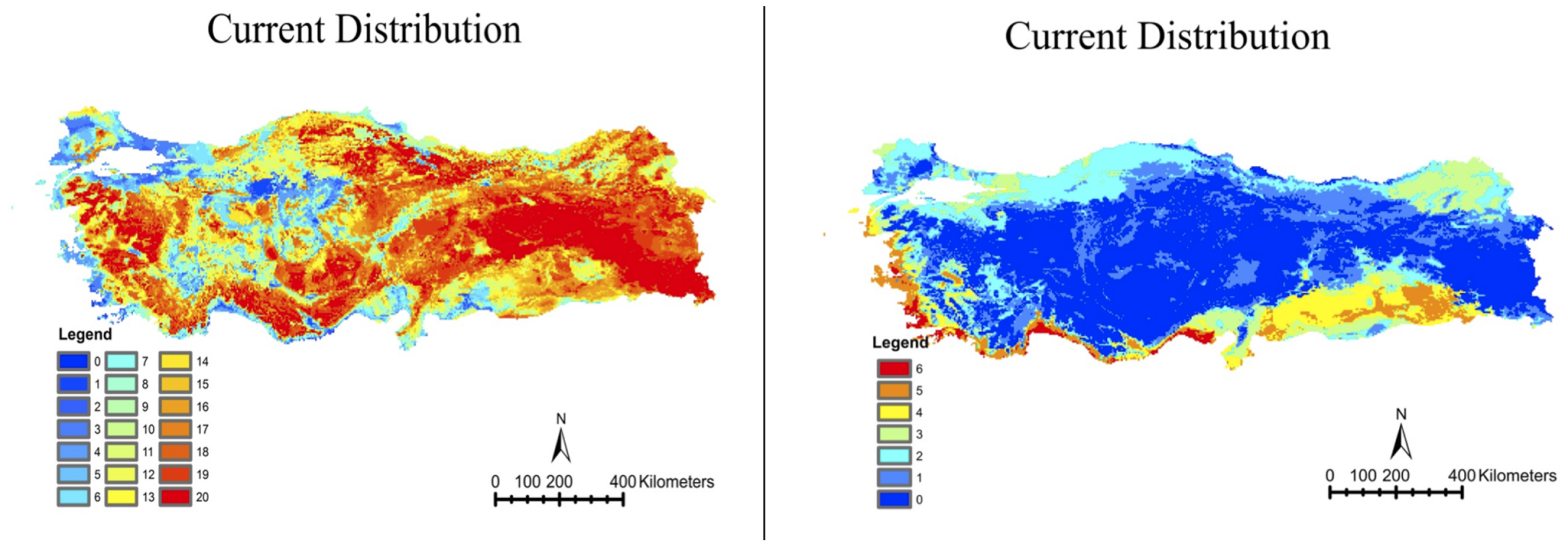
Maps of the current distribution patterns of the a. 20 resident b. nine summer migrant species analyzed in our study.

The combined species richness maps for the resident and migratory species are given in Figure 1a and Figure 1b, respectively. In our data set, resident species richness was relatively lower in coastal areas and in central Anatolia. For migratory species, no presence was predicted in Central Anatolia and Eastern Anatolia Regions. However, it is likely that the absence of widespread species in these regions could be due to a relative lack of observers.

**Figure 2:**
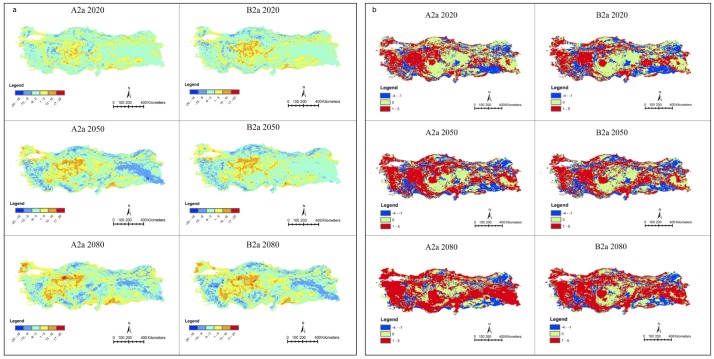
Maps of species richness change (negative values indicate grids with species lost; positive values indicate grids with species gained) under different time periods and scenarios for a. resident species b. migratory species.

The projected changes in the richness of our study species over time are shown in Figure 2a and Figure 2b. In both scenarios, greatest declines in our study species were predicted in Eastern Anatolia by 2080 (Figure 2a). Until 2020, in both scenarios our model projected a small increase in study species richness, mostly in the Central Anatolia, Southeastern Anatolia, and partly in the Marmara and the Eastern Black Sea Regions. In Eastern Anatolia, a decrease in species richness was the dominant pattern. This decrease was mostly low (a decline of 0 to 8 species analyzed) in 2020. In the A2a scenario, this decline reached 15 species in 2050, but a recovery is expected to happen in this area in 2080. In scenario B2a, no loss of study species is expected in eastern Anatolia between 2020 and 2050, whereas the areas where study species are expected to be lost increased in eastern Anatolia by 2080. In the Marmara Region (northwestern Turkey), the gain and loss maps showed similar patterns in both the A2a and B2a scenarios. In 2020, there was a projected increase around the Marmara Sea of up to nine species. This projected increase spread through the Thrace Region incrementally, reaching up to 16 species by 2050. In the Aegean and the Mediterranean Regions, a gain of up to nine species is expected in the coastal areas during each time period, but in the inland parts of these regions, losses are expected, with up to eight species by 2020 and up to 15 species by 2050. The Central Anatolian and Southeast Anatolian regions, on the other hand, show a projected increase in the number of species in all time periods and under both scenarios. In 2020, this increase is expected to approach nine species. The areas with higher numbers of species increase westward and southward from Central Anatolia, reaching the eastern part of the Aegean and northern part of the Mediterranean regions by 2050. By 2080, this increase is expected to reach 17–20 species in certain areas.

By 2020, there is a projected gain of up to five species of migratory songbirds in the west of the country, whereas species loss is expected in the Southeastern Anatolia and Eastern Black Sea regions (Figure 2b). The eastern part of the Central Anatolia region, which currently does not host any of the summer migratory species we analyzed (Figure 1b), is not expected to show any significant modifications in species ranges. Under both scenarios, a relative increase is projected for the Black Sea Region by 2050, and for central Anatolia by 2080.

**Figure 3:**
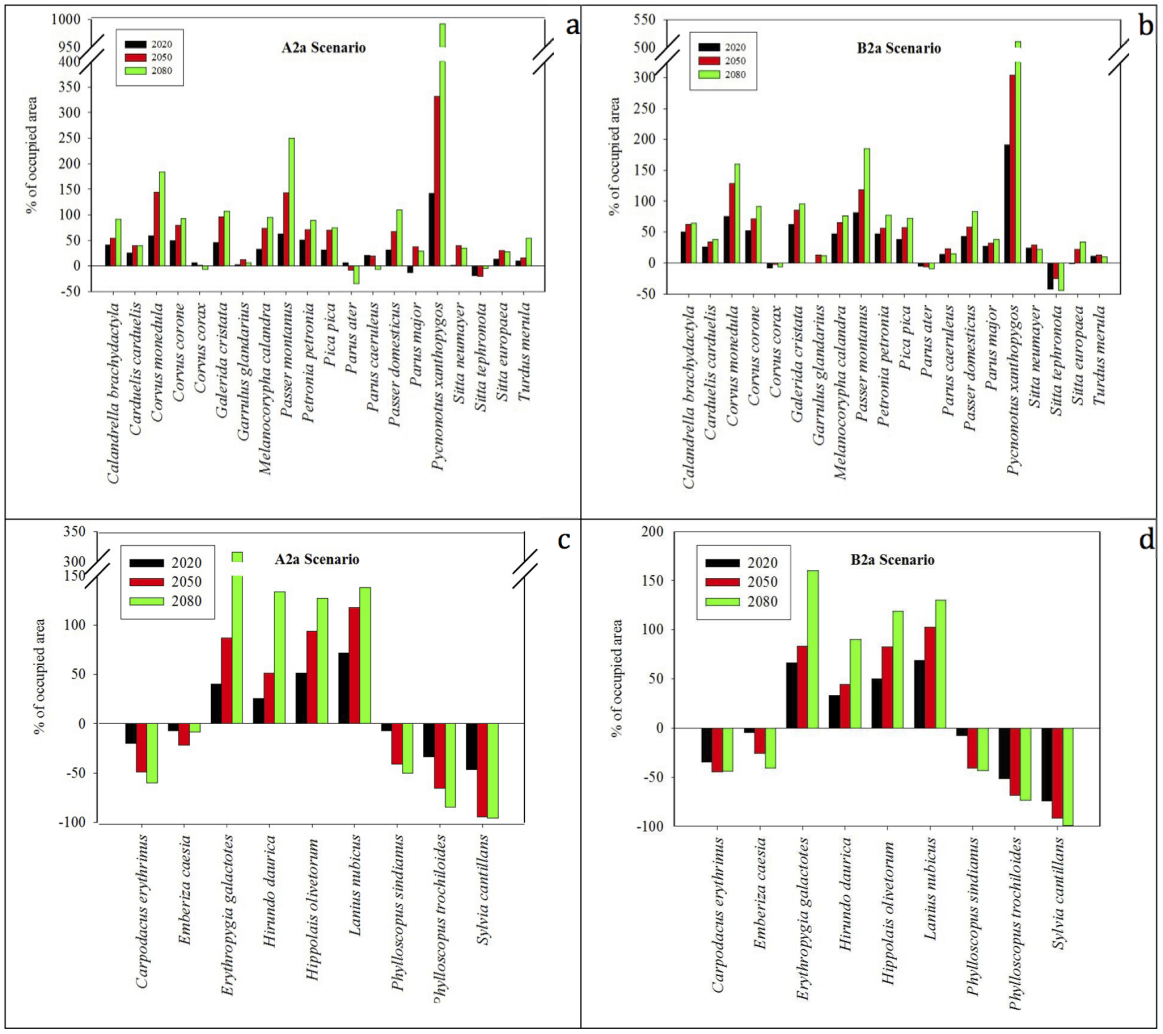
Expansion/contraction pattern of the species by years in scenario a. resident species, A2a b. resident species, B2a c. migratory species, A2a d. migratory species, B2a (the bars above zero point indicate expansion of species). Bar heights were determined by calculating the total grid area of a species’ predicted presence for both the current and future time periods. Bars above or below the x-axis represent the expansion or contraction in the distribution area of a particular species, respectively.

**Figure 4:**
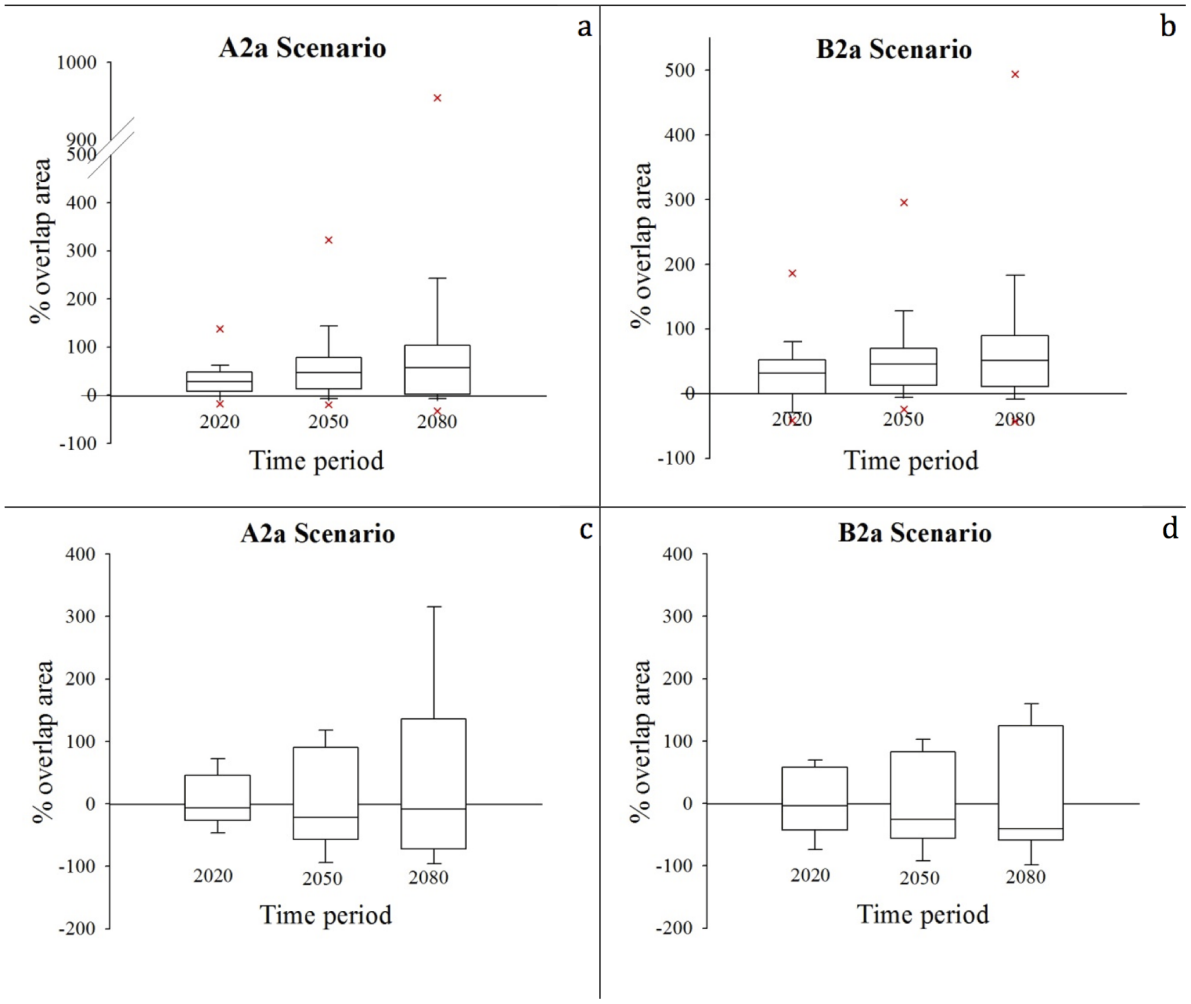
Proportional range shift area between projected models and the current potential distribution (the vertical bar indicates maximum and minimum values registered for a resident bird species within each group; outliers show the portion of maximum expansion shift for a species). **a.** under scenario A2a for resident species. **b.** under scenario B2a for resident species. **c.** under scenario A2a for migratory species. **d.** under scenario B2a for migratory species.

Model predictions with Maxent and different scenarios showed high variability in the projection of resident species’ range shifts, with some species projected to lose and most species projected to gain suitable habitat, depending on the scenario (Figures 3 and 4). For our study species, future suitable locations in Turkey were within the dispersal distance of maximum likelihood based on the model and natal dispersal values in Barbet-Massin *et al.* [42]. Therefore, we assumed full dispersal for our study species. When the ensemble model results were analyzed separately, we found that in scenario A2a only one species (Eastern Rock Nuthatch *Sitta tephronota*) was consistently predicted to have a smaller distribution, and 14 of the 20 species were consistently predicted to have larger distributions. On the other hand, in scenario B2a, three of the 20 species (Common Raven, Coal Tit, and Eastern Rock Nuthatch) were consistently predicted to have smaller distributions and 16 of the 20 species were consistently predicted to have larger distributions. The remaining species showed increases and decreases depending on the time period and scenario. For example, Great Tit and Wood Nuthatch were predicted to have smaller distributions until 2020 and then increase their distribution by 2080 in A2a and B2a scenarios, respectively. On the other hand, European Goldfinch was predicted to have a larger distribution until 2050 and then decrease its distributional area in 2080, under scenario A2a. Four migratory species, Common Rosefinch, Cretzschmar's Bunting, Mountain Chiffchaff and Greenish Warbler are expected to lose more than half of the area they currently use for breeding (Figures 4a, b). The expected loss was most significant for Subalpine Warbler, which was predicted to lose most of its breeding habitat through 2020 and 2050. By 2080, the species is expected to lose about 96% and 99% of its breeding area in scenarios A2a and B2a, respectively.

**Figure 5:**
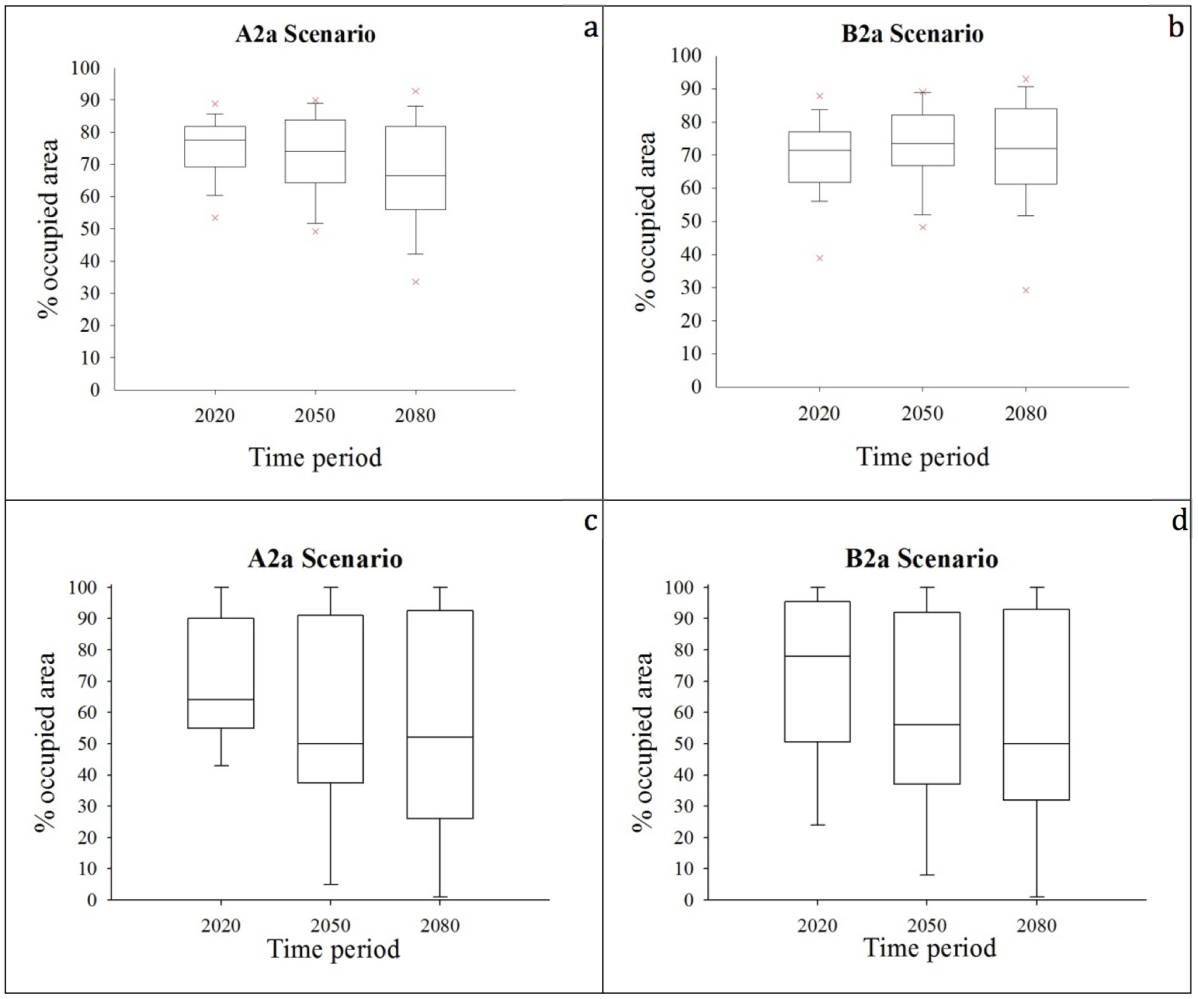
The change of occupied area throughout the time periods in relation to area currently occupied by species (the vertical bar indicates maximum and minimum values registered for a bird species within each group). **a.** under scenario A2a for resident species. **b.** under scenario B2a for resident species. **c.** under scenario A2a for migratory species. **d.** under scenario B2a for migratory species.

Increases in the ranges of resident species are expected, with the median values of 28%, 47%, and 56% in A2a scenario and 31%, 45%, and 51% in B2a scenario by the years 2020, 2050 and 2080, respectively (Figure 5a, 5b). The outlier is the species with the greatest range expansion, White-spectacled Bulbul. Decreases in the ranges of migratory species are expected, with median values of −7%, −22%, and −9% in A2a scenario and −4%, −26%, and −41% in B2a scenario by the years 2020, 2050 and 2080, respectively (Figure 5c, 5d). The outliers in the graph show the species with the highest degree of range contractions.

### Species Turnover

**Figure 6:**
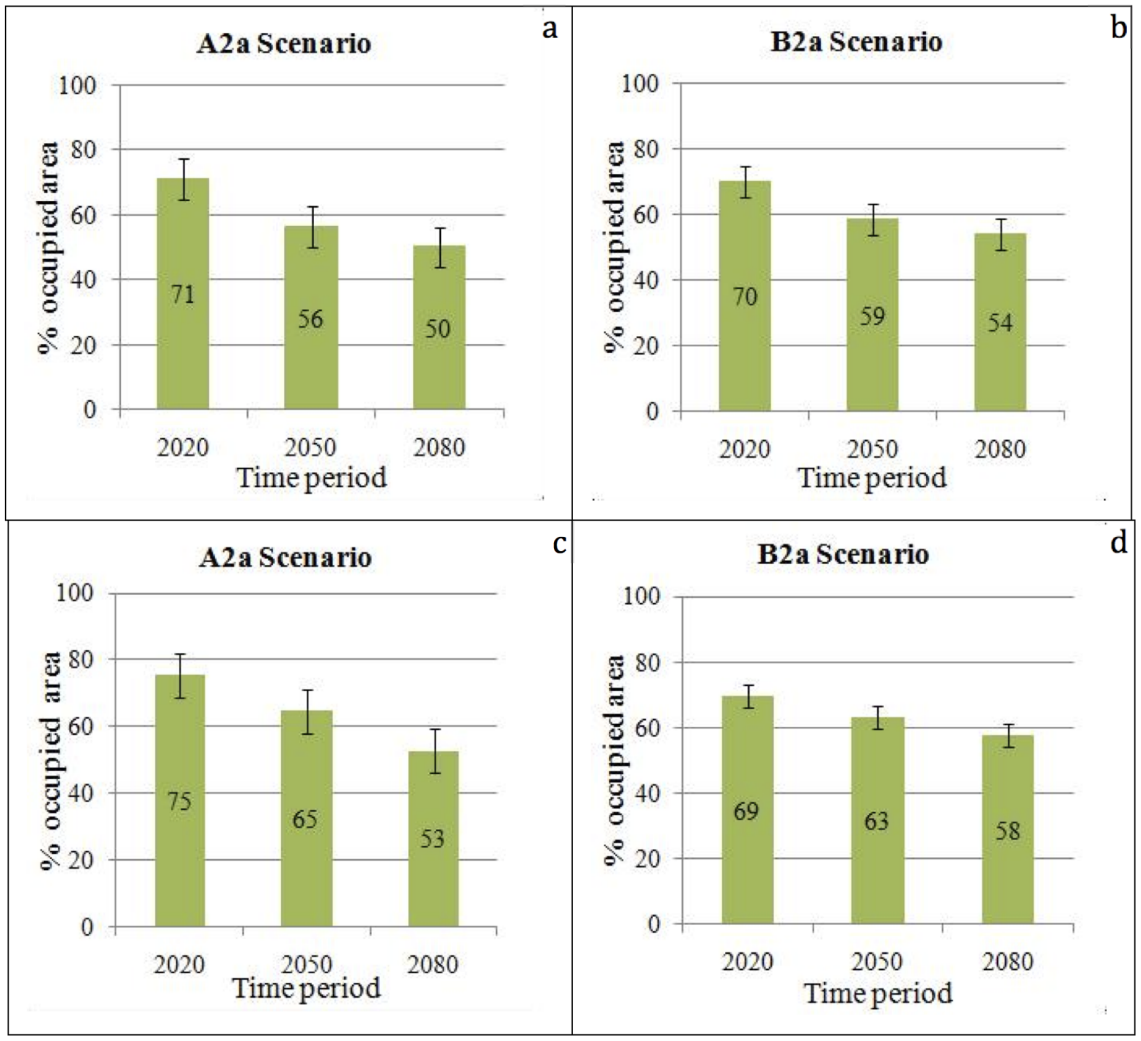
Average percentage of occupied area that is expected to host the same species in all timelines (the vertical lines indicate standard deviation). **a.** under scenario A2a for resident species. **b.** under scenario B2a for resident species. **c.** under scenario A2a for migratory species. **d.** under scenario B2a for migratory species.

Among different species, the extent of the current occupied area that will still be occupied in the future varies according to the two scenarios (Figure 6). The average percentage of the areas that are expected to continue to host the same species in each time period compared to the previous one (2080 compared to 2050, 2050 to 2020 and 2020 to current) are given in Figures 6a and 6b for resident species, and in Figures 6c and 6d for migratory species. Under both scenarios, areas that will still be occupied by the same resident species gradually decrease over the four timelines. In the scenarios A2a and B2a, only 50% and 54% of the areas would keep the same resident species in all time periods at the end of the study period, respectively (Figures 6a, 6b), whereas 53% and 58% of the areas in the scenarios A2a and B2a, respectively, are expected to be occupied by the same migratory species during the same period (Figures 6c, 6d).

The calculations for centroids show that species are likely to be displaced to a greater extent for the models created under scenario A2a rather than scenario B2a (Table 5). The species that exhibited relatively large shifts between the current time period and 2080 (as an average of A2a and B2a scenarios), included Great Tit (a ∼468 km shift in centroids), Rock Sparrow (∼328 km), White-spectacled Bulbul (∼479 km), and Eastern Rock Nuthatch (∼210 km) among resident species. Among migratory species, greatest shifts were observed in Rufous-tailed Scrub-robin (∼396 km) and Subalpine Warbler (∼419 km). These large-scale changes do not necessarily mean that a species will completely migrate out of Turkey; for instance, the range of White-spectacled Bulbul is expected to shift from southern to northern Anatolia with warming temperatures and changing vegetation cover. On the other hand, Anatolia is expected to become completely unsuitable as breeding habitat for Subalpine Warbler, a migratory species. Among the resident species in our study, European Goldfinch (∼29 km), Hooded Crow (∼35 km), Eurasian Jackdaw (∼35 km), House Sparrow (∼40 km), and Wood Nuthatch (∼25 km) show relatively smaller average range shifts in terms of centroid movement. Among the migratory species analyzed, Cretzschmar's Bunting (∼45 km) and Greenish Warbler (∼54 km) showed the smallest shifts in their breeding distributions.

**Table 5:** The range shift of individual species for the different time periods based on centroid calculations (km).

	A2a-2020	A2a-2050	A2a-2080	B2a-2020	B2a-2050	B2a-2080
*Calandrella brachydactyla*	68.75	64.88	116.77	44.30	27.61	96.40
*Carduelis carduelis*	7.21	4.86	47.17	6.87	25.20	10.43
*Corvus corax*	6.20	13.02	101.23	29.96	12.86	57.90
*Corvus corone*	19.31	10.18	48.81	24.14	34.96	21.08
*Corvus monedula*	3.17	28.29	44.32	3.59	33.34	25.17
*Galerida cristata*	18.04	71.79	143.98	42.26	45.89	100.63
*Garrulus glandarius*	57.24	16.29	64.41	55.77	20.07	8.59
*Melanocorypha calandra*	38.92	28.37	106.90	46.58	10.44	75.28
*Parus ater*	86.85	174.40	191.15	129.38	166.95	191.09
*Parus caeruleus*	35.23	61.51	47.31	44.83	53.60	52.31
*Parus major*	34.32	32.39	936.18	45.40	14.04	0.65
*Passer domesticus*	8.27	12.71	78.94	11.27	23.93	0.41
*Passer montanus*	100.07	167.90	156.18	174.13	120.69	153.97
*Petronia petronia*	138.86	268.30	334.12	147.26	280.92	322.86
*Pica pica*	50.13	54.70	146.98	65.50	27.52	97.34
*Pycnonotus xanthopygos*	84.34	328.15	543.63	185.60	303.82	416.16
*Sitta europea*	8.86	13.82	8.16	19.12	18.04	41.33
*Sitta neumayer*	47.75	24.36	22.24	40.19	61.12	41.55
*Sitta tephronota*	19.85	7.34	218.15	84.44	62.63	201.92
*Turdus merula*	50.53	53.93	17.53	54.33	63.58	44.07
*Carpodacus erythrinus*	33.22	35.61	102.68	76.40	46.22	53.67
*Emberiza caesia*	84.40	101.29	57.18	111.06	101.22	33.44
*Erythropygia galactotes*	139.52	275.08	457.56	216.96	265.27	334.60
*Hippolais olivetorum*	22.97	101.08	156.53	64.83	68.35	134.17
*Hirundo daurica*	170.75	141.17	120.69	176.81	111.40	118.48
*Lanius nubicus*	91.63	76.50	122.64	74.44	76.61	93.15
*Phylloscopus sindianus*	251.27	47.49	98.27	52.58	29.38	92.54
*Phylloscopus trochiloides*	15.05	44.37	55.09	98.64	48.80	52.17
*Sylvia cantillans*	71.67	43.67	751.44	116.08	128.98	87.77

Finally, in terms of species richness, almost no change was observed in the area with no modeled presence (i.e. all study species absent) for resident species (1% for current vs 0% for future periods). This may be due to the increase in the area, which had six to ten species (15% for current, around 28% for future periods). Nevertheless, in migratory species there was a decrease in no modeled presence (from 44% for current to 18–20% total decrease for future periods), indicating an overall increase in the distribution of the migratory species in our study. Reductions (from 73% to 65%) were also predicted in the areas that include 15 to 20 of the resident study species and five to nine migratory species (from 6% to 3%).

## Discussion

Both climate scenarios have similarities with regard to the potential impacts of climate change upon resident and summer migrant breeding birds in Turkey. For the resident species, seasonality of temperature, seasonality of precipitation, annual mean temperature, annual precipitation, and precipitation of the driest month were identified as the most important bioclimatic variables that affect the presence or absence of the selected songbird species. Mean temperature and precipitation of the warmest quarter, and seasonality of precipitation were the most important bioclimatic variables for summer migratory species. These results are in line with past findings, which indicate that temperature, food and water source availability contribute significantly to songbird migration and survival [3], [43], [44].

Most of the species analyzed within the scope of this study exhibit a clear tendency for overall displacements towards the north. However, the magnitude of these displacements varies amongst species and between the two climate scenarios examined. The greatest range shifts expected, on the range of several hundred kilometres, are for the Great Tit, Rock Sparrow, White-spectacled Bulbul, and Eastern Rock Nuthatch among the resident species, and Rufous-tailed Scrub-robin and Subalpine Warbler among the migratory species we studied. These results analyzed in conjunction with the data on species’ expansions might provide some additional insights. For instance, Great Tit, while increasing its range about 50%, exhibits a large shift in its centroid (∼468 km), indicating that its numbers will deteriorate in its current range. Hence displacement might be a threat for Great Tit, although its range is not necessarily decreasing. The case is even worse for Eastern Rock-nuthatch whose range is predicted to decrease by about 50%, with a coupled big shift between its centroid locations.

Our model results suggest that some songbird species will show different patterns than other songbirds and not all studied species are likely to experience niche reductions. This could be due to multiple reasons. First, the reaction of individual species to climate change depends on their adaptive potential and also the current and potential future rate of change in climate, which are not the same between regions [44]. Second, Turkey is a key area for geographical variation of bird populations, as the birds of the Western Palearctic found in various habitats such as European deciduous forests, Mediterranean scrub and wetlands, Arabian semi-desert, Caucasian Mountains and central Asian steppes all meet in Turkey [27], [28]. These subpopulations are observed all over the country, which spans a diverse range of climate. For example, one of the study species, Crested Lark *Galerida cristata*, has five subspecies recorded in Turkey: *G. c. meridionalis* is observed in the Aegean and the Mediterranean regions, *G. c. subtaurica* in the Central Anatolia and Eastern Anatolia regions, *G. c. caucasica* in the coastal zone of the Black Sea, *G. c. cinnamomina* around Hatay Province, and *G. c. zion* in southeastern Turkey. All of these subspecies’ inhabit areas that have almost completely different bioclimatic features. If our objectives in this study were to consider and identify possible dispersal changes at the subspecies level, we would have probably observed more significant changes than what we detected at the species level.

Furthermore, in migratory species, there is a decrease in no modelled presence (from 44% for current to 18–20% for future periods). A decrease is also predicted in the areas which contain 15 to 20 resident species (from 73% to 65%) and five to nine migratory species analysed (from 6% to 3%). This indicates clear and predictable range shifts for the migratory species we studied, as these birds look for specific bioclimatic features either for breeding or wintering [45]. Therefore even small changes in local climate scenarios are expected to affect most of the common migratory species we studied.

Two (under B2a) or three (under A2a) of the 20 resident and five of the nine migratory studied species in both scenarios are predicted to experience contractions in their total occupied area. Furthermore, nearly 42% and 46% of future modelled presence for resident and migratory species, respectively, will occur in areas that do not contain the same species today. These results show that songbird species will need to shift their ranges in order to adapt to climate change and the phenological changes are likely to cause changes in distributions, as observed in other species [5], [9], [20]. An assumption of our analyses was that every species could migrate independently between different areas. Hence, if an area is bioclimatically suitable for a species, the species is considered to be able to migrate into that new area with 100% dispersal ability. On the other hand, if the climatic conditions are not ideal for a species, a species is expected to immediately abandon a certain area. However, areas that fulfil all the necessary climatic conditions for a species might not meet other crucial conditions for a species’ existence, such as food and roosts, or these areas may not have suitable vegetation due to the presence of cities, fields, and other human-dominated habitats. Hence, our models project the best-case scenarios in terms of species distributions. These major range shifts may be impossible if there is no suitable habitat available in species’ future ranges, a likely scenario given the rapid expansion of humanity’s footprint in Turkey at the expense of wildlife [25].

The potential reductions in the extent of some species’ breeding distributions will put a number of species at risk of extinction in the region. Moreover, some species’ potential future distributions do not overlap with their current distributions. This will lead to significant population decreases and put additional species at risk of extinction, especially if the dispersal capabilities of these species are limited. These include migratory species such as Cretzschmar's Bunting, Mountain Chiffchaff, Greenish Warbler, and Subalpine Warbler. On the other hand, potential expected distributions of some of the species examined indicate dramatic expansions. Those species that extend their breeding distributions and colonize new areas could pose competitive risks for already resident species that show more or less stable distributions. These risks include competition for high quality habitat and nesting sites, and reduced breeding success due to increased competition and insufficient food resources [20]. Therefore, while the sensitive species will be affected negatively, the dominant and expanding ones are likely to benefit from these new climatic conditions.

Our results demonstrate that climate change in Turkey will not affect every species in the same manner. Overall, there is not a significant decrease in the area occupied by the resident species we analysed, but there is substantial species turnover. The maximum decrease in area occupied for a resident species we analysed is projected for Eastern Rock Nuthatch, with a decrease of 44% in scenario B2a. However, the distributions of three summer migratory species, Common Rosefinch, Greenish Warbler, and Subalpine Warbler, are expected to decline by more than 50% by 2080. Among these species, Subalpine Warbler is expected to suffer the most drastic range reductions; our models indicate >90% decline in its current breeding area by the year 2050 in both scenarios and >99% by 2080 in scenario B2a. If roosting and habitat limitations are considered, the actual decrease in occupied areas will likely be higher than the models suggest, so the breeding populations of the Subalpine Warbler in Turkey may go extinct by 2080. On the other hand, three resident species (Eurasian Jackdaw, House Sparrow, and White-spectacled Bulbul) and four migratory species (Rufous-tailed Scrub-robin, Red-rumped Swallow, Olive-tree Warbler, Masked Shrike) are project to have more than 150% and 100% increases in their distributions, respectively. The highest increase in a resident species is expected for White-spectacled Bulbul (992%), and in a migratory species, for Rufous-tailed Scrub-robin (315% in scenario A2a). Both species are found in the typical hot Mediterranean climate in southern Turkey, and with increasing temperatures, these species are expected to expand their ranges in Turkey four to eleven-fold.

Our study has shown that there will be extensive turnover across Turkey in the species composition of bird communities, with some species declining and others expanding their ranges. The outputs of our species models show that summer migratory species whose breeding areas are in the northern (Common Rosefinch, Mountain Chiffchaff, and Greenish Warbler*)* and western (Subalpine Warbler) parts of Turkey will progressively lose suitable climate space. Alternatively, species that have restricted breeding areas in the southern parts of the country will have the potential to expand their ranges to the northwestern parts of the study area. It is also likely that some Middle Eastern species currently not found in Turkey may expand their ranges into Turkey in response to climate change. The consequences of climate change in terms of movement between different geographic regions are, therefore, variable.

Due to the lack of sufficient data, our study was able to model the potential future distributions of only 29 of Turkey’s 151 known bird passerine species. Approximately 360 bird species out of 470 are regularly found in Turkey and among these are many European bird species that are expected to change their distributions due to climate change [46]. Due to the low numbers of amateur and professional ornithologists in Turkey, most bird species do not have sufficient distributional data to enable the analyses we have undertaken. An urgent priority is to increase ornithological research in Turkey and make better use of data collected by amateur but experienced birdwatchers [47], whose numbers are steadily increasing in the country. Our study is an important first step for similar future investigations that will have access to bigger data sets in Turkey and in the region.

At the country level, some of the modelled species are expected to be highly sensitive to climate change. For these species, more detailed monitoring is required to verify the predictions of our models. Conservation management and regulations may need to be strengthened for these species, in order to continue their existence in Turkey. Increasing and restoring potential habitats by expanding protected areas will help in maintaining the populations of some species. Translocation may be another method to decrease the vulnerability of these species. However, translocations are unlikely to be feasible except in a few cases, and for the species in our study area with predominantly western and northern distributions, the disappearance of climatically suitable habitat will pose a major risk of extinction.
